# Model of a Support Vector Machine to Assess the Functional Cure for Surgery of Intermittent Exotropia

**DOI:** 10.1038/s41598-019-38969-x

**Published:** 2019-06-06

**Authors:** Yanli Liu, Chungao Liu, Wei Zhang, Xia Chen, Kanxing Zhao

**Affiliations:** 1 Baodi Clinical College, Tianjin Medical University, 8 Guangchuan Road, Tianjin, 301800 China; 2 Tianjin Baodi Hospital, 8 Guangchuan Road, Tianjin, 301800 China; 3 State Key Laboratory of Hydrology-Water Resources and Hydraulic Engineering, Hohai University, Nanjing, 210098 China; 4 Tianjin Eye Hospital, 4 Gansu Road, Tianjin, 300020 China; 5 Tianjin Key Laboratory of Ophthalmology and Visual Science, 4 Gansu Road, Tianjin, 300020 China; 6 Tianjin Eye Institute, 4 Gansu Road, Tianjin, 300020 China

**Keywords:** Ocular motility disorders, Applied mathematics

## Abstract

In this paper the optimum timing for the postoperative functional cure of basic intermittent exotropia is explored based on support vector machine (SVM). One hundred and thirty-two patients were recruited in this prospective cross-sectional study with 6 months of follow-up. Examinations included angle of deviation, central and peripheral fusion, controllability, and near and distance stereopsis. Influencing factors of postoperative alignment and stereopsis were analyzed with a chi-squared test and univariate and multivariate logistic regression analyses. At 6 months post-operation, there were 84 successful procedures for the angle of deviation, with 4 overcorrections and 44 undercorrections. The success rate was 63.6%. The angle of deviation on postoperative day 1 was the only significant associated factor. One hundred and thirty patients had normal near stereoacuity, 60 had normal distance stereoacuity according to a Functional Visual Analyzer assessment, and 108 had normal stereoacuity as assessed by the Frisby Davis Distance (FD2) stereotest. The age of onset and preoperative distance stereoacuity with FD2 were the influencing factors of postoperative distance stereopsis restoration. The accuracy of this method of SVM was 82.1%. The angle of deviation for distance on postoperative day 1 was the only significant factor that correlated with alignment at 6 months post-operation, and the model of SVM was useful to determine the optimal time of the postoperative functional cure.

## Introduction

Intermittent exotropia (IXT) is a common type of childhood strabismus. Its onset usually occurs between 3 and 6 years, however, it might be detected much later in childhood. Binocular function is often disturbed by exotropia. In the phoric phase, the patient might have excellent stereopsis. With a gradual increase in the tropic frequency, patients might show a decrease or loss of stereopsis because of the large regional suppression of the temporal retina and anomalous retinal correspondence^1^. Surgery is an effective method for the treatment of IXT^2^. The goals are to restore alignment and to preserve or restore binocular function. The critical point is the establishment of postoperative stereopsis. A popular intervention criterion includes the reduction or loss of stereoacuity, or deteriorating control. However, the threshold of intervention remains poorly defined^3^. The present prospective cross-sectional clinical study examined preoperative factors that might contribute to postoperative success. Additionally, a support vector machine (SVM) model was designed to show the optimal time for surgical intervention based on the preoperative factors and provide valuable clinical guidance.

## Subjects and Methods

This study adhered to the principles of the Declaration of Helsinki and was approved by the ethics committee of the Tianjin Eye Hospital, PRC(YKLL-2015-8-21). The study was registered with the Chinese Clinical Trial Registry (http://www.chictr.org/en/; ChiCTR-OOC-15006997). Patients fulfilling the eligibility criteria were recruited from the pediatric eye clinic at Tianjin Eye Hospital, China, from September 2015 to March 2016. For each child, written informed consent was obtained from all the parents and children prior to enrollment.

Inclusion criteria were as follows:

1. Age 5–35 years.
2. Basic exotropia (defined disparity between deviations for distance and near was not more than 10 prism diopters (PD) before and after 60 min monocular patching); vertical deviation was <5 PD.
3. Distance exodeviation between 15 and 50 PD without an A/V pattern.
4. Best-corrected visual acuity was normal for age.
5. Visual acuity difference between the eyes was not greater than two lines.

Exclusion criteria were as follows:

1. Gestational age <34 weeks.
2. Birth weight <1500 g.
3. Prior strabismus surgery or convergence exercises or patching.
4. Developmental delay, systemic illness, syndromes or learning disability.
5. Hyperopia or myopia greater than 5.00 D spherical equivalent in either eye, or astigmatism greater than 2.00 D.
6. Anisometropia (spherical aberration ≥1.5 D or cylindrical aberration ≥1.0 D).
7. Coexisting ocular pathology.

All patients underwent ophthalmological examination by the same ophthalmologist preoperatively and postoperatively with follow-ups of 1 day, 6 weeks, 3 months and 6 months. Measurements were obtained using spectacles that corrected the patient’s refractive errors.

1. Recorded the following data: age, gender, race, gestational age, birth weight, family history of strabismus, age at onset, course of disease, and squinting in sunlight.
2. Refractive error in both eyes by cyclorefraction was converted to the spherical equivalent.
3. Control ability was determined by the Revised Newcastle Control Score.
4. Peripheral fusion (Worth’s Four-Dot test at 1/3 m) and central fusion (Worth’s Four-Dot test at 5 m) were assessed.
5. Distance stereoacuity was determined (Frisby Davis Distance [FD2] Stereotest, Stereotest Ltd; Functional Visual Analyzer [FVA], Stereo Optical Co. Inc, America).
6. Near stereoacuity was determined (Titmus circles, Stereo Optical Co. Inc, America; Frisby, Richmond products, Co. Inc, America).
7. Deviation at distance fixation (6 m) and near fixation (1/3 m) was determined by a prism alternate cover test.

All surgeries were performed by three of the authors (KXZ, WZ, XC) according to consistent surgical formulae (Table 1). Participating clinicians managed children according to consistent normal clinical criteria.

**Table 1:** Surgical formulae in this study.

Deviation (PD)	ULR (mm) (n = 48)	BLR-rec (mm) (n = 42)	R&R (mm) (n = 42)
18	7.5	—	—
20	8	—	—
25	—	5	4/3
30	—	6	5/4
35	—	6.5	6/4
40	—	7	7/5
45	—	7.5	7.5/5.5
50	—	8	8/6

Note that: PD is prism diopter; ULR-rec is unilateral lateral rectus recession; BLR-rec is bilateral lateral rectus recession; R&R is unilateral lateral rectus recession combined with medial rectus resection.

Surgical outcomes were deemed successful according to the angle of deviation at 6 months post-operation (esophoria/tropia ≤5 PD or exophoria/tropia ≤10 PD), recurrent (exotropia >10 PD) or overcorrected (esophoria/tropia >5 PD). The latter two were deemed failures. Near stereoacuity ≤60 arc second was deemed normal. Distance stereoacuity ≤20 arc second with FD2 and ≤40 arc second with FVA was deemed normal.

### Statistical analyses

Analyses were performed using SPSS version 19 (IBM Corporation, NY), and values of *p* < 0.05 were considered statistically significant. Influencing factors of postoperative eye position and stereoscopic restoration were analyzed with a chi-squared test and univariate and multivariate logistic regression analyses. The model of a support vector machine (SVM) was used to find a clear cut-off point for functional cures after surgery.

## Results

### Demographics and preoperative characteristics of the patients

One hundred and thirty-two patients were recruited, including 74 males and 58 females. The median age was 10 (6, 12.25) years. The distance deviation was 30 (20, 40) PD, and near deviation was 30 (25, 41.25) PD. The preoperative characteristics are shown in Tables 2–4.

**Table 2:** Preoperative Newcastle Control Score (Unit: person).

scores	Newcastle home Control Score	Newcastle office Control Score for distance	Newcastle office Control Score for near
0	0	6	26
1	40	8	22
2	42	28	36
3	50	90	48
Total	132	132	132

**Table 3:** Preoperative Worth’s Four-Dot test (Unit: person).

5 m	33 cm			Total
	4 lights	5 lights	3 or 2 lights	
4 lights	14	0	0	14
5 lights	14	6	0	20
3 or 2 lights	60	6	32	98
Total	88	12	32	132

**Table 4:** Preoperative and postoperative stereopsis test (Unit: person).

Stereopsis	Titmus		Frisby		FVA		FD2	
	preop	postop	preop	postop	preop	postop	preop	postop
Normal	100	130	100	130	10	60	60	108
Decline	22	2	22	2	38	42	24	18
Negative	10	0	10	0	84	30	48	6
χ^2^ Value	20.31		20.31		64.35		44.55	
*p* Value	0		0		0		0	

Note that: FVA is Functional Visual Analyzer; FD2 is Frisby Davis Distance.

### Postoperative characteristics of the patients

**Figure 1:**
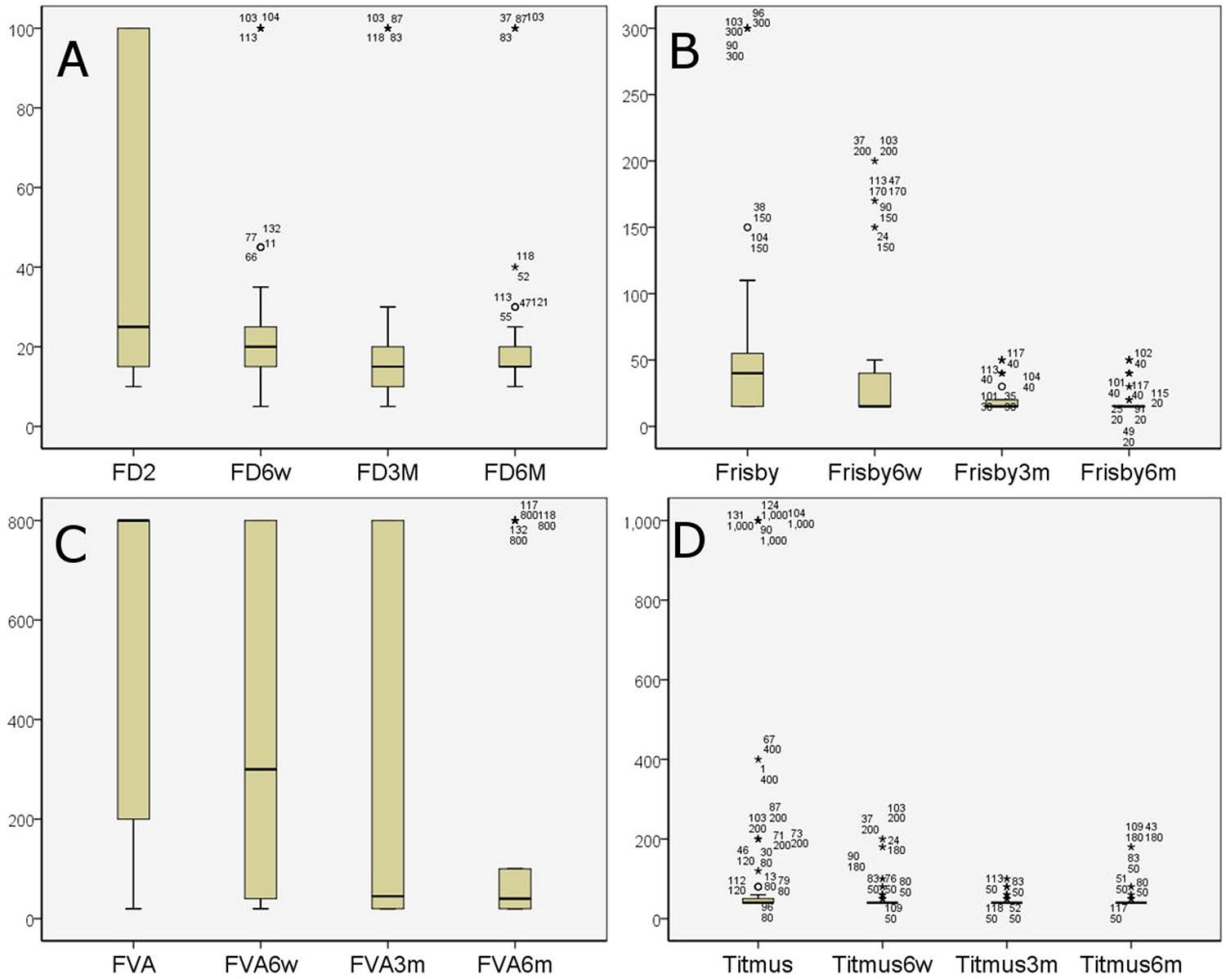
Preoperative and postoperative stereopsis test at 6-weeks, 3-months, and 6-months. The Y-axis represents the stereoacuity with arcsecond as the unit. Note that: FVA is Functional Visual Analyzer; FD2 is Frisby Davis Distance.

The postoperative angles of deviation at 1 day, 6 weeks, 3 months and 6 months are shown in Table 5. At 6 months after surgery, deviation success was observed in 84 persons, undercorrection was found in 44 persons, and overcorrection was found in 4 persons. The success rate was 63.6%. The restoration of stereopsis is shown in Tables 3 and 6 and Fig. 1. The patients with intermittent exotropia had good near stereoacuity both preoperatively and postoperatively. One hundred and thirty persons had normal near stereopsis (98.48%). The two abnormal patients were both overcorrected (shown in Table 6). The preoperative distance stereopsis of most patients was damaged. Distance stereopsis of most patients could be recovered postoperatively at 3 months (shown in Fig. 1). Table 6 shows that the effect of deviation on stereopsis with Titmus, Frisby, and FD2 were not statistically significant (*p* > 0.05), and the effect of deviation on stereopsis with FVA was statistically significant (*p* > 0.05).

**Table 5:** Postoperative angle of deviation of the patients.

Postoperative alignment (PD)	1 day	6 weeks	3 months	6 months
Success (−5~−10)	94 (71.2%)	94 (71.2%)	88 (66.7%)	84 (63.6%)
Undercorrection (>−10)	16 (12.1%)	34 (25.8%)	38 (28.8%)	44 (33.3%)
Overcorrection (>+5)	22 (16.7%)	4 (3%)	6 (4.5%)	4 (3%)

**Table 6:** Deviation and stereopsis test at 6 months post-operation.

Deviation	Titmus		Frisby		FVA		FD2	
	normal	abnormal	normal	abnormal	normal	abnormal	normal	abnormal
Success	84	0	83	0	46	38	74	10
Failure	46	2	47	2	14	34	34	14
Total	130	2	130	2	60	72	108	24
χ^2^ Value	0.08		0.08		4.036		2.01	
*p* Value	0.775		0.775		0.045		0.156	

Note that: FVA is Functional Visual Analyzer; FD2 is Frisby Davis Distance; Success is (esophoria/tropia ≤5 PD or exophoria/tropia ≤10 PD); Failure is (exotropia >10 PD or esophoria/tropia >5 PD).

The angle of deviation at distance on postoperative day 1 was the only factor showing a significant association in the results of the univariate logistic regression analysis (*p* = 0.037, <0.05) (shown in Table 7). It was also the only significant risk factor for postoperative 6-month restoration of distance stereopsis with FD2 (*p* = 0.048, <0.05). The risk factors affecting postoperative 6-month restoration of distance stereopsis with FVA were age of onset and preoperative distance stereopsis with FVA and FD2, besides realignment with univariate logistic regression analysis (shown in Table 8). The above three factors were analyzed with a multivariate logistic regression analysis among patients with alignment at 6 months post-operation. The age of onset (*p* = 0.023, <0.05) and the preoperative distance stereopsis with FD2 (*p* = 0.036, <0.05) were statistically significant. The two factors had a cross effect, so it was difficult to find a clear cut-off point for surgical intervention by conventional statistical methods.

**Table 7:** Univariate logistic regression analysis of deviation at 6 months postop.

Parameters	Deviation at 6 months postop		
	Coefficient b	Wald value	*p* value
gene	0.693	1.699	0.192
close one eye in sunlight	0.421	0.652	0.419
age	−0.06	2.308	0.129
age of onset	−0.067	1.65	0.199
course of disease	−0.049	0.727	0.394
family history	−0.211	0.072	0.789
refractive error	−2.74	2.134	0.144
total NCS	−0.103	0.696	0.404
home NCS	−0.176	0.319	0.572
clinic distance NCS	−0.073	0.054	0.817
clinic near NCS	−0.218	0.926	0.336
distance deviation	0.01	0.314	0.575
near deviation	−0.006	0.116	0.733
worth four-dot test (2 m)	0.109	0.078	0.78
worth four-dot test (33 cm)	0.725	5.653	0.051
Titmus	0	0.575	0.448
Frisby	0.003	1.174	0.279
FVA	0	0.152	0.697
FD2	0	0.003	0.955
surgical procedures	0.056	0.036	0.85
deviation at postop- 1 day	−0.093	4.371	0.037

Note that: If the eye position is success, the value is 0. If the eye position is overcorrected or uncorrected, the value is 1. FVA is Functional Visual Analyzer; FD2 is Frisby Davis Distance; NCS is Newcastle Control Score.

**Table 8:** Univariate logistic regression analysis of postop-stereopsis with FVA.

Parameters	FVA at 6 months postop		
	Coefficient b	Wald value	*p* value
gene	0.581	0.859	0.354
close one eye in sunlight	0.090	0.019	0.890
age	−0.085	3.679	0.055
age of onset	−0.203	5.250	0.022
course of disease	0.031	0.223	0.637
family history	1.460	1.525	0.217
refractive error	0.056	0.052	0.819
total NCS	0.164	0.972	0.324
home NCS	0.249	0.427	0.513
clinic distance NCS	0.637	1.784	0.182
clinic near NCS	0.072	0.058	0.810
distance deviation	−0.008	0.135	0.713
near deviation	−0.012	0.327	0.567
worth four-dot test(33 cm)	1.041	3.105	0.078
worth four-dot test(2 m)	0.779	2.223	0.123
Titmus	0.000	0.049	0.825
Frisby	0.004	1.252	0.263
FVA	0.002	3.958	0.047
FD2	0.021	6.375	0.012
surgical procedures	−0.552	2.098	0.147

Note that: If the stereoacuity is normal, the value is 0. If the stereoacuity is abnormal, the value is 1. FVA is Functional Visual Analyzer; FD2 is Frisby Davis Distance; NCS is Newcastle Control Score.

SVM can solve the nonlinear classification problem by projecting low-dimensional nonlinear space into high-dimensional linear space through a kernel function. Therefore, the problem of neural network structure selection and a local minimum can be avoided. SVM is a small sample learning method; it does not involve probability measurement or the law of large numbers. Therefore, unlike the existing statistical methods, the general classification problem is greatly simplified. The goal of SVM is to establish the optimal hyperplane of feature space partitioning. The final decision function is determined by only a few support vectors. The computational complexity depends on the number of support vectors, not the dimension of the sample space. Therefore, the “dimensional disaster” was avoided. The model of SVM is a classification method that determines the best plane/line to classify the subjects with the highest accuracy. Therefore, the SVM was used to consider the influence of the above two factors. The formula was derived by the traditional method^4^.

Forty-two patients were randomly selected from the 84 patients with success in deviation at 6 months post-operation as a training sample, and the remaining 42 cases were used to verify the accuracy of the method. The SVM was used for calculations.

Input: age, preoperative stereoacuity with FD2; output: 0 and 1 (0 for abnormal postoperative stereoacuity with FVA, 1 for normal postoperative stereoacuity).

**Figure 2:**
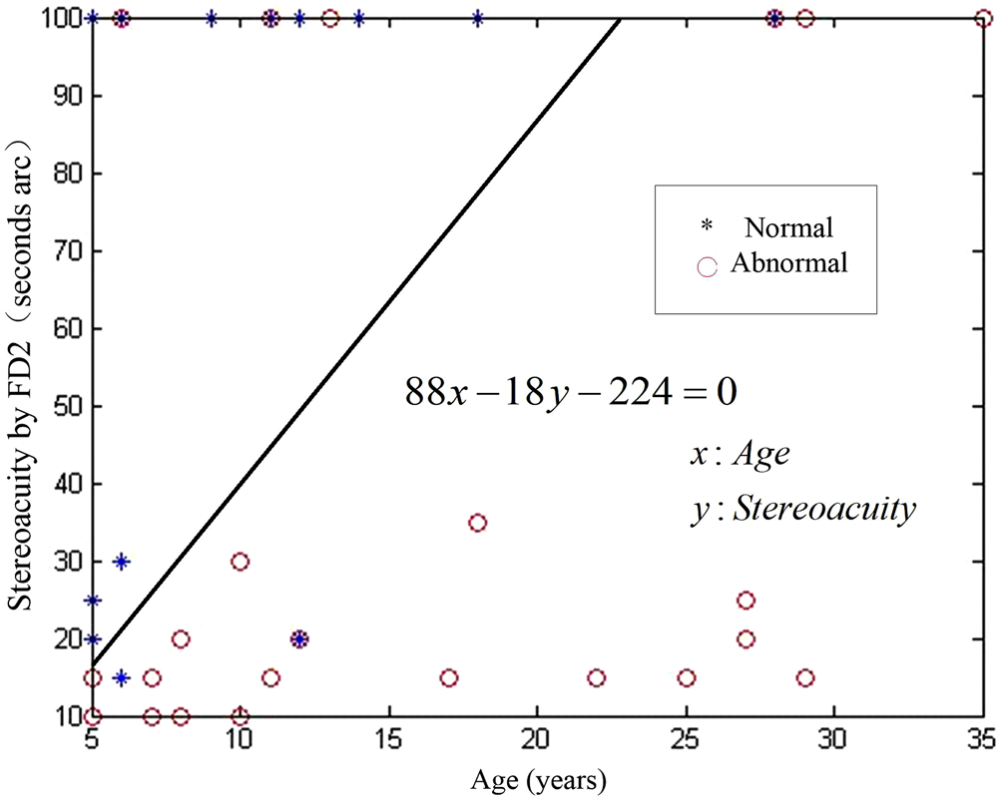
The dividing line of normal and abnormal distance stereopsis by Frisby Davis Distance (FD2).

The dividing line for normal and abnormal is shown in Fig. 2, and its equation was 88*x*-18y-224 = 0, where *x* represented the age of the sample and *y* represented preoperative distance stereoacuity with FD2. When the result of the equation was more than zero, it was marked as normal, otherwise it was marked as abnormal. The accuracy of the method was 82.1%.

## Discussion

IXT is a common type of strabismus in children. The angle of deviation gradually increases. The purpose of surgery is not only cosmetic but also for reconstruction of binocular visual function. The factors affecting the surgical outcomes are wide ranging, such as age at surgery^5–7^, preoperative angle deviation and refractive errors^8–10^, fusion function and stereopsis^11,12^, and early postoperative alignment^13^, that the optimal time of surgical intervention was still not clear. Jung EH, *et al*.^10^ reported the surgical results of 39 adult patients with at least 1 year follow-up. The rate of surgical success, overcorrection, and recurrence were, respectively, 72%, 18%, and 10%. The alignment at postoperative week 1 was the only significant factor associated with surgical results, and the best early postoperative alignment was <10 PD of esotropia. Ruttum MS, *et al*.^14^ offered a similar conclusion with 6 months of follow-up and 63% surgical success. 33Kim HJ, *et al*.^15^ reported that the angle of deviation at distance on postoperative day 1 was the only significant factor that correlated with surgical outcomes at 2 years post-operation. The conclusions of this study were consistent with the above findings. The angle of deviation at distance on postoperative day 1 was the only factor associated with the last angle of deviation measured at 6 months. This was mainly determined by the operator’s preoperative examination of the patient’s maximum angle of deviation and surgical design.

Xue, F *et al*.^16^ reported that patients with intermittent exotropia had good near stereoacuity both preoperatively and postoperatively. The preoperative distance stereopsis of most patients was damaged. Distance stereopsis of some patients could be recovered postoperatively. In this study, the conclusion was similar to the previous study. The postoperative near stereopsis was normal except for two overcorrected patients at the last follow-up. The postoperative distance stereopsis was normal in 54.5%, measured by FVA, and 81.8%, measured by FD2. The time of surgery was the key point when distance stereopsis was cured after surgical treatment.

There was no improvement in postoperative stereopsis of some patients who never appeared with esotropia after surgery. Persistent suppression in the central retina was the main mechanism^17^. A previous study showed that retinal suppression occurred before stereoscopic damage^18^. The sizes of the stereograms varied, which were projected to different ranges of the retina. The range of retinal suppression could lead to various results among stereotests, because the size of their stimulus was different. For example, the FD2’s stimulus was larger than FVA’s, so the stereoacuity of some patients with IXT using FD2 was better than those using FVA. Saxena R, *et al*.^19^ suggested that a high grade of preoperative stereoacuity was the significant factor in the achievement of normal stereoacuity postoperatively at 6 months. Singh A, *et al*.^20^ reported that a distance stereoacuity worse than 20 arc sec was an indication for surgical intervention. It is well known that the younger patients with strabismus were more likely to form retinal suppression. Age was one of the most important factors effecting stereopsis. In this study, the age and preoperative distance stereoacuity with FD2 were statistically significant for normal stereoacuity according to FVA at 6 months post-operation. The two factors had an interactive influence on postoperative stereoscopic restoration. It was difficult to find the cut-off point of the optimal time for surgical intervention through traditional statistical methods. The model of a support vector machine intuitively showed the cut-off point of distance stereoacuity by FD2 in every age. The distance stereoacuity using FVA was normal at 6 months post-operation with alignment, if the surgery was done before the cut-off point.

One limitation of this study was that it included patients age 5 to 35 years, so the findings could not be directly applied to children under 5 years or adults older than 35 years. Additionally, the number of subjects was small and different surgical methods were involved.

## Conclusion

The angle of deviation at distance on postoperative day 1 was the only significant factor that correlated with alignment at 6 months post-operation. The age of onset and preoperative distance stereoacuity were significant associated factors of postoperative restoration of normal stereopsis. The method of SVM was an effective method to show the clear cut-off point for optimum timing of surgery in every age group.

## Supplementary information

Author_List_Changes_Approval_form
