# Supplementary material for: Model of a Support Vector Machine to Assess the Functional Cure for Surgery of Intermittent Exotropia
Source: Sci Rep. 2019 Jun 6;9:8321. doi: 10.1038/s41598-019-38969-x (PMC6554402; doi:10.1038/s41598-019-38969-x)
Supplement: Supplementary file 1 — Author_List_Changes_Approval_form [file 41598_2019_38969_MOESM1_ESM.docx]

In accordance to Nature Publishing Groups Authorship Policy we agree to change the authors of the manuscript as indicated below.

**NAME OF JOURNAL**: Scientific Reports

**TITLE OF MANUSCRIPT**: **Model of a Support Vector Machine to Assess the Functional Cure for Surgery of Intermittent Exotropia**

**MANUSCRIPT NUMBER**: SREP-18-14726A

**CORRESPONDING AUTHORS NAME:** Kanxing Zhao

**PREVIOUS AUTHOR NAMES:**

Yanli Liu, Chungao Liu, Kanxing Zhao

**UPDATED AUTHOR NAMES:**

Yanli Liu, Chungao Liu, Wei Zhang, Xia Chen, Kanxing Zhao

**CHANGE TO AUTHOR LIST:** Wei Zhang and Xia Chen were added as the 3rd and 4th authors as they performed operations, so change Kanxing Zhao as the 5th author.

| **Print Name** | **Signature** | **Date** |
| --- | --- | --- |
| Yanli Liu | 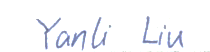 | 08/30/2018 |
| Chungao Liu | 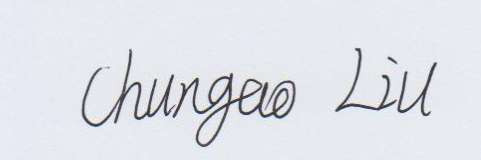 | 08/30/2018 |
| Wei Zhang | 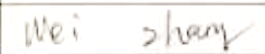 | 08/30/2018 |
| Xia Chen | 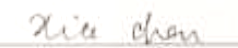 | 08/25/2018 |
| Kanxing Zhao | 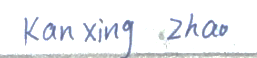 | 08/30/2018 |
|  |  |  |
|  |  |  |

I was not proficient in the operation of the manuscript submission system of your journal for the first time, I didn’t know how to save it at that time. It took me a long time to fill it out from the front to the end. At that time, I did not know the email address of Wei Zhang and Xia Chen. This is a required item. If I did not fill in their email address, I could not submit my manuscript. After i deleted their message, I submitted successfully. After revising the manuscript this time according to the opinions of the reviewers, I feel that my paper is more advanced than the original manuscript, and may be employed by your journal. As the important participants and contributors of this clinical study, we have to add them.

The following table is an itemised list of the contributions of each author to the manuscript.

| Author | Contributions |
| --- | --- |
| Yanli liu | -conceived and designed and carried out the experimental protocol;  -ophthalmological examination preoperatively and postoperatively with follow-ups of 1 day, 6 weeks, 3 months and 6 months;  -collected the data;  -wrote the first draft of the manuscript;  -confirmation of the final manuscript;  -submission of papers |
| Chungao Liu | -the analysis of data;  -designed Model of Support Vector Machine;  -confirmation of the final manuscript; |
| Wei Zhang | -performed operations for patients in the subject according to consistent -surgical formulae ;  -confirmation of the final manuscript; |
| Xia Chen | -performed operations for patients in the subject according to consistent surgical formulae ;  -confirmation of the final manuscript; |
| Kanxing Zhao | -conceived and designed and carried out the experimental protocol；  -performed operations for patients in the subject according to consistent surgical formulae ;  -reviewed and revised the manuscript;  -confirmation of the final manuscript; |
